# The Alternative Splicing Mutation Database: a hub for investigations of alternative splicing using mutational evidence

**DOI:** 10.1186/1756-0500-1-3

**Published:** 2008-02-26

**Authors:** Jason M Bechtel, Preeti Rajesh, Irina Ilikchyan, Ying Deng, Pankaj K Mishra, Qi Wang, Xiaochun Wu, Kirill A Afonin, William E Grose, Ye Wang, Sadik Khuder, Alexei Fedorov

**Affiliations:** 1 Program in Bioinformatics and Proteomics/Genomics, University of Toledo Health Science Campus, Toledo, Ohio 43614, USA; 2 Dept. of Medical Microbiology and Immunology, University of Toledo Health Science Campus, Toledo, Ohio 43614, USA; 3 Dept. of Basic Pharmaceutical Sciences, South Carolina College of Pharmacy, University of South Carolina, Columbia, South Carolina 29208, USA; 4 Department of Biological Sciences, Bowling Green State University, Bowling Green, Ohio 43403, USA; 5 College of Engineering, University of Toledo, Toledo, Ohio 43606, USA; 6 Dept. of Medicine, University of Toledo Health Science Campus, Toledo, Ohio 43614, USA

## Abstract

**Background:**

Some mutations in the internal regions of exons occur within splicing enhancers and silencers, influencing the pattern of alternative splicing in the corresponding genes. To understand how these sequence changes affect splicing, we created a database of these mutations.

**Findings:**

The Alternative Splicing Mutation Database (ASMD) serves as a repository for all exonic mutations not associated with splicing junctions that measurably change the pattern of alternative splicing. In this initial published release (version 1.2), only human sequences are present, but the ASMD will grow to include other organisms, (see Availability and requirements section for the ASMD web address).

This relational database allows users to investigate connections between mutations and features of the surrounding sequences, including flanking sequences, RNA secondary structures and strengths of splice junctions. Splicing effects of the mutations are quantified by the relative presence of alternative mRNA isoforms with and without a given mutation. This measure is further categorized by the accuracy of the experimental methods employed. The database currently contains 170 mutations in 66 exons, yet these numbers increase regularly.

We developed an algorithm to derive a table of oligonucleotide Splicing Potential (SP) values from the ASMD dataset. We present the SP concept and tools in detail in our corresponding article.

**Conclusion:**

The current data set demonstrates that mutations affecting splicing are located throughout exons and might be enriched within local RNA secondary structures. Exons from the ASMD have below average splicing junction strength scores, but the difference is small and is judged not to be significant.

## Background

About 50% of mammalian genes exhibit alternative splicing (AS) – the production of multiple mRNA isoforms from the same gene, often in a tissue- or development stage-specific manner. In humans, the number of different types of expressed mRNA appears to be two to three times higher than the total number of genes [1,2]. The regulation of alternative splicing is a very intricate process which involves the interaction of dozens of spliceosomal proteins with a great variety of short sequence motifs inside exons and introns. These regulatory motifs are known as exonic splicing enhancers (ESEs), exonic splicing silencers (ESSs), intronic splicing enhancers (ISEs), and intronic splicing silencers (ISSs) [1,3]. Pre-mRNA secondary structures are also important players in the regulation of alternative splicing (see review [4]).

Significant progress in understanding AS has been achieved in experimental research that characterized a number of splicing enhancers and silencers [5-9] and also in several bioinformatics approaches for computational inference of ESEs and ESSs [10-18]. Despite this progress, one cannot predict a tendency to alternative splicing from genomic data. A set of mutations known to be associated with alternative splicing effects (reviewed by [19,9]) provides valuable raw material for a broad range of studies aiming to elucidate mechanisms of spliceosomal regulation.

In order to advance this area of research, we have created the Alternative Splicing Mutation Database (ASMD) – a collection of human exon sequences with short (1–6 nucleotides) internal mutations that change the balance of alternatively spliced mRNA isoforms or cause the appearance of new mRNA isoforms. The ASMD includes only those mutations that change exonic enhancers and silencers and does not encompass those that change splice sites (deletion of existing splice junctions or creation of novel junctions). The ASMD is manually curated such that each entry is meticulously verified with published literature describing the influence of the mutation on alternative splicing. This information has been converted into a novel parameter, termed "Splicing Effect" or SE value. The SE value lies within a range of [-1, +1] and reflects the effect of a mutation on an observed change in the pattern of alternative splicing. In the case of exon skipping, for example, SE = -1 means that a mutation causes 100% skipping of the constitutive wild-type exon. The database also contains an evaluation of the accuracy of the experimental techniques underlying the SE value for each mutation. The ASMD web site allows for the display of an array of information on every database entry, including splice site strength scores and putative RNA secondary structures.

There already exist many AS-related databases dating back to 1999. They are all important for their contributions to the understanding of alternative splicing. Nevertheless, the ASMD's focus on mutations sets it apart from each of these efforts. Analyzing a high-quality, curated database of mutations could conceivably lead to the identification of novel mediators of splicing and give a unique evaluation of the strength of splicing enhancers and silencers.

## Construction and content

The Alternative Splicing Mutation Database (ASMD) version 1 uses a relational database (MySQL) to accurately represent the relationships between the core entities: genes, mutations, and splicing effects. In addition, the database incorporates annotation information in the form of putative local RNA secondary structures, splice sites and their consensus value and log-odds scores. Finally, references, notes, and depositor information has been included in the database to facilitate long-term growth and collaboration.

All wild-type sequences are derived from the human Exon-Intron Database, most from version 35p1, some from version 36p1 [20,21]. Both wild-type and mutant exon sequences for each mutation are stored in the sequences table. Mutant sequences are generated by the incorporation of published mutations into the wild-type sequence. All sequences are then properly annotated in the sequence feature table. Splice site scores are calculated using both the consensus value and log-odds methods, as described in Zhang *et al*. 2005 [10]. Local RNA secondary structures are predicted using the RNALfold utility from the Vienna RNA package, version 1.6.1 [22], with default parameters and a window size of 30 nucleotides. Only structures with a minimum free energy (mfe) of -10.0 kcal/mol or lower were loaded into the database.

Explanations of "Splicing Effect" (SE) values, determination of SE accuracy levels, and other parameters are provided in the glossary, which is accessible from the home page.

## Utility

### ASMD dataset and browsing features

**Figure 1:**
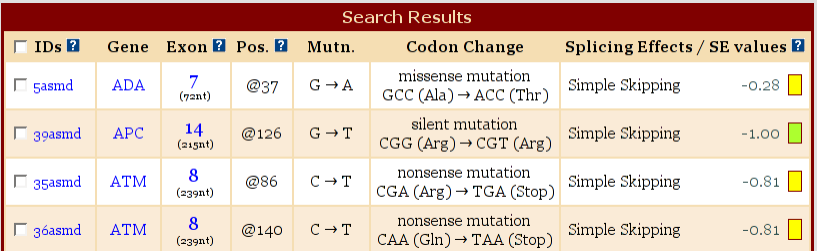
**ASMD search results example**. This screen capture shows the first four entries from the default search (i.e. no restrictions). The fields in blue are links to further views of the data. The colored boxes next to the SE values code the accuracy of the data. Explanations for accuracy levels and for fields marked with the blue and white question mark icon are available on the glossary page on the web site.

**Figure 2:**
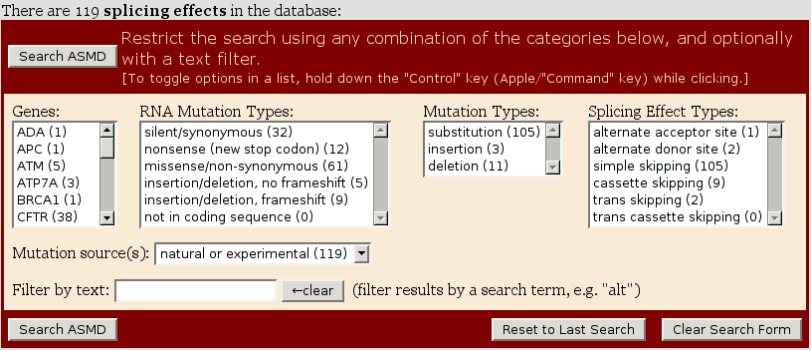
**ASMD search form**. This screen capture shows the complete search form. Note that the numbers in parentheses next to each category represent a count of the splicing effects, not the mutations in that category.

**Figure 3:**
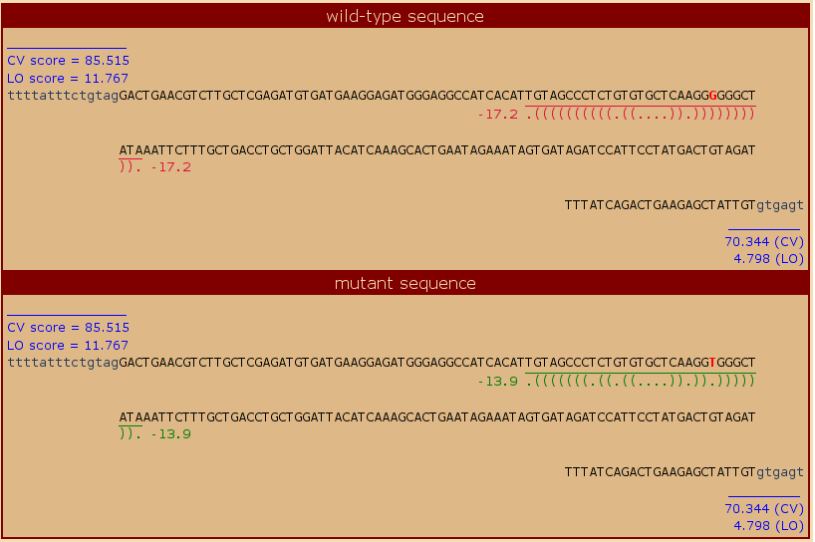
**ASMD sequence comparison display**. This screen capture shows the sequence comparison display from the detailed mutation view (obtained by clicking on the ASMD ID in the search results). The mutation position is highlighted in red. The splice site strength scores are shown in blue along with the flanking intronic sequences. The putative local RNA secondary structure ("fold") is shown along with its minimum free energy (mfe) value. Note how the mutation disrupts the base-pairing in the stem of the fold, substantially reducing the strength of the fold.

The ASMD web site consists of three main sections: a home page, a search page, and a public depositions area. The home page is the starting point and provides connections to all parts of the site. The search page is used for locating mutations and splicing effects in the database. It contains the complete search form at the bottom of the page. Figure 1 shows four entries of the ASMD and Figure 2 shows the search form. Views of mutations as well as sequences of genes and exons are accessible from this page. Figure 3 shows part of the detailed mutation view, which is accessible through the ASMD identifier. The public depositions area contains instructions and forms for the submission of mutations, published references, and notes.

The ASMD sequence data is available in FASTA format from a link on the home page. The informational lines in the file contain characteristics of the gene, the mutation, and the associated splicing effect(s) while the sequence contains the wild-type exon in which the mutation occurs. An explanation of the FASTA-formatted data is available on the web site.

### ASMD usage

We expect researchers interested in understanding alternative splicing (AS) will use ASMD in their investigations in two complementary ways. By searching in ASMD for genes, exons, and mutations of interest, it is hoped that researchers may be able to link observed AS isoforms with particular mutations and their correlated sequence features, such as putative RNA secondary structures. And depositing new mutations and their splicing effects into ASMD, we foresee researchers interactively improving the power and utility of this resource.

Because ASMD fundamentally differs from other AS databases in its focus on the effects of mutations, it functions differently from other existing databases. Instead of receiving an exhaustive list of observed alternative splicing events for a gene or exon of interest, a researcher using ASMD can expect to find a curated list of small mutations that are correlated with alternative splicing effects, as documented in the literature. This will enable researchers to craft experiments accordingly, to either avoid duplication of effort or to further understanding of AS regulation, both at specific loci and in general.

### Future development

The main task for the ASMD is to expand its dataset to cover all known mutations that affect splicing. The process of culling examples from the literature continues and new mutations are being added monthly. We are in the process of updating our sequences to build 36.1 of the human genome. Updates for tools and calculations will be performed every six months as the database grows.

Currently, entries are limited to mutations inside human exons. In future releases we wish to expand the domain to include mutations inside introns and in other mammalian species. Accordingly, we plan to expand our analysis of RNA secondary structures into all parts of pre-mRNA including introns and splicing junctions. Once a sufficient variety of exonic and intronic mutations is obtained for a given gene, a new display will be added to capture the effects of multiple mutations on alternative splicing. Where data exists, this display could also capture the synergistic effects of multiple mutations, a phenomenon already documented in the literature [23].

### ASMD data analysis

**Figure 4:**
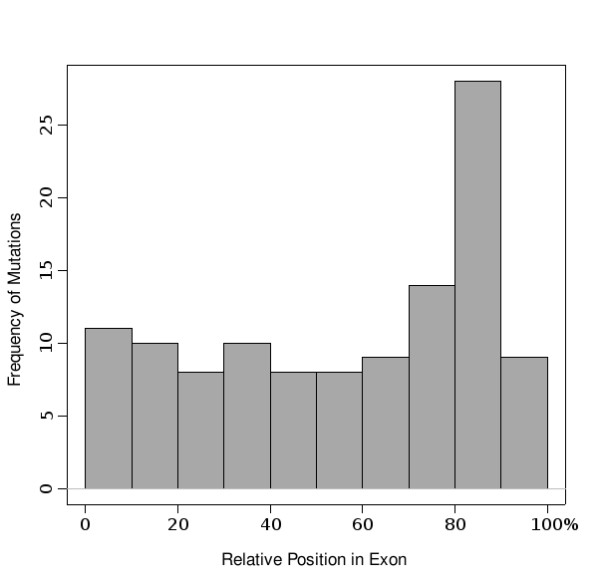
**Distribution of the relative position of mutations within exons**. This histogram shows a generally even distribution of mutation positions. The x-axis represents the relative position of each mutation within its exon, calculated as percentage of exon length. The lone spike around the 80–90% position is an abundance of mutations from the experimental mutagenesis data on exon #10 of the CFTR gene.

**Figure 5:**
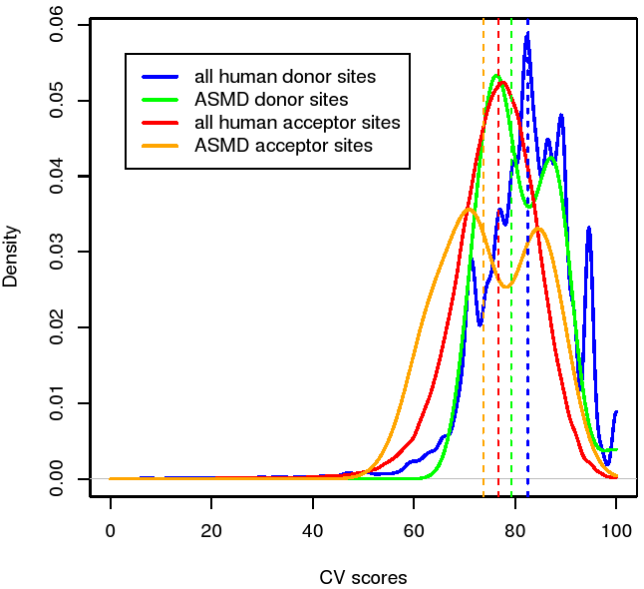
**Splice site strength scores for wild-type ASMD exons vs. "all" human exons**. These density curves (smooth-line histograms) represent the relative strength of splice sites as determined by the consensus value (CV) method. Donor and acceptor sites are considered separately. The vertical dashed lines indicate the median values. The sample of 193,995 human splice sites was obtained from the Exon-Intron Database's dEID file, version hs35p1, and was confined to the purged sample of 11,316 non-redundant human genes referred to in the Methods section.

ASMD version 1.1 data demonstrate that mutations affecting splicing are located throughout exons and are not restricted to the ends near splice junctions (see Fig. 4). An analysis of 34 unique exons in the database shows that their splice site strengths have median scores *slightly *below those of all human exons (see Fig. 5). The difference is small, however, compared to the standard deviation and is judged not to be significant.

ASMD version 1.1 data suggest that mutations affecting splicing are somewhat enriched within local RNA secondary structures (LRSS). Further, those mutations within LRSS may specifically avoid loops and may have a special preference for "dangling ends" (bases adjacent to helices in free ends and multi-loops).

We first observed that there are no strong LRSS in wild-type exons with mutations conferring a *positive *splicing effect (i.e. decreased skipping). The only putative LRSS in this subset of exons has a calculated minimum free energy (mfe) of -9.0 kcal/mol. None of the splice-affecting mutations in that exon (exon #10 of the *CFTR *gene) coincide with this putative secondary structure.

The ASMD version 1.1 dataset contains 91 mutations conferring a *negative *splicing effect (i.e. increased skipping). There is a greater prevalence of putative LRSS in the exons carrying these mutations. 11% of the bases in these exon sequences are within putative LRSS. The number of observed mutations within LRSS compared to random expectation represents an average enrichment of 21% for ten different combinations of folding parameters. The mutations that occur within putative LRSS of -10 kcal/mol or stronger are ASMD IDs 12, 25, 46, 47, 49, 52, 60, 73, 112, and 116.

We also examined the presence of splice-affecting mutations in stems and loops, where stem positions were further broken down into base-pairings, bulges, and dangling ends. Over the same set of parameter combinations, the average percentage of mutations within loops, base-pairings, bulges, and dangling ends is 5, 40, 35, and 20%, respectively.

We judge the current data to indicate a slight trend toward splice-affecting mutations occurring within the stems of local RNA secondary structures, specifically at the "dangling ends." However, subsequent Monte Carlo simulations with the appropriate statistical tests (Chi-squared or Fisher exact) revealed none of these trends to be statistically significant (α = 0.1) with the current data. Statistical evaluation of a larger data set should be performed to confirm or reject these hypotheses.

## Conclusion

The ASMD represents a collection of small internal exonic mutations, not associated with splicing junctions, that change the pattern of alternative splicing. The ASMD web site allows a user to explore the connections between mutations and features of their surrounding sequences, including putative RNA secondary structures and strengths of splice junctions. As the database grows, so too will the predictive power of associated tools and our understanding of the mechanisms regulating alternative splicing. By creating the ASMD public deposition area, we encourage the scientific community to participate in the development of the database.

## Methods

All calculations were performed using the ASMD dataset version 1.1, which contained 119 mutations in 37 exons. It is implemented using MySQL and PHP on GNU/Linux.

A set of 20,433 sequences of human intron-containing protein coding genes from the Exon-Intron Database [20,21] was purged of all homologs (≥50% protein identity) and of genes with multiple repetitive domains (more than 4 repeats of the same 5-aa fragment) to obtain a reduced set of 11,316 human genes. This sample of non-redundant human genes is available from our web page  as file "HS35.1.purge3.dEID".

## Availability and requirements

**Project name: **The Alternative Splicing Mutation Database

**ASMD project home page: **

**Operating system(s): **Platform-independent

**Programming Language: **PHP

**Other requirements: **a modern web browser (with CSS and JavaScript support)

**License: **GNU GPL v3

**Restrictions to use by non-academics: **None (not applicable under GPL)

## List of abbreviations

AS: Alternative splicing; ASMD: The Alternative Splicing Mutation Database; ESE: Exonic splicing enhancer; ESS: Exonic splicing silencer; ISE: Intronic splicing enhancer; ISS: Intronic splicing silencer; LRSS: Local RNA secondary structure; SE: Splicing effect.

## Competing interests

The authors declare that they have no competing interests.

## Authors' contributions

The ASMD resource was conceptualized and developed by JMB and AF. PR, II, YD, PKM, QW, XW, KAA, WEG, YW, JMB, and AF were responsible for the biological input for this project, collecting and processing the mutational datasets, and obtaining and interpreting results. SK was responsible for all statistical analyses. AF supervised the project, provided guidance, and wrote the draft. All authors have read and approved the final manuscript.

## References

[B1] Stamm S, Ben-Ari S, Rafalska I, Tang Y, Zhang Z, Toiber D, Thanaraj TA, Soreq H (2005). Function of alternative splicing. Gene.

[B2] Wu JY, Havlioglu N, Yuan L, Meyers RA (2004). Alternatively spliced genes. Encyclopedia of Molecular Cell Biology and Molecular Medicine.

[B3] Wang Z, Xiao X, Van Nostrand, Burge CB (2006). General and specific functions of exonic splicing silencers in splicing control. Mol Cell.

[B4] Buratti E, Baralle FE (2004). Influence of RNA secondary structure on the pre-mRNA splicing process. Mol Cell Biol.

[B5] Tian H, Kole R (1995). Selection of novel exon recognition elements from a pool of random sequences. Mol Cell Biol.

[B6] Coulter LR, Landree MA, Cooper TA (1997). Identification of a new class of exonic splicing enhancers by *in vivo* selection. Mol Cell Biol.

[B7] Liu HX, Zhang M, Krainer AR (1998). Identification of functional exonic splicing enhancer motifs recognized by individual SR proteins. Genes Dev.

[B8] Schaal TD, Maniatis T (1999). Selection and characterization of pre-mRNA splicing enhancers: identification of novel SR protein-specific enhancer sequences. Mol Cell Biol.

[B9] Valentine CR (1998). The association of nonsense codons with exon skipping. Mutat Res.

[B10] Zhang XH, Leslie CS, Chasin LA (2005). Computational searches for splicing signals. Methods.

[B11] Zhang XH, Kangsamaksin T, Chao MS, Banerjee JK, Chasin LA (2005). Exon inclusion is dependent on predictable exonic splicing enhancers. Mol Cell Biol.

[B12] Stadler MB, Shomron N, Yeo GW, Schneider A, Xiao X, Burge CB (2006). Inference of splicing regulatory activities by sequence neighborhood analysis. PLoS Genet.

[B13] Down TA, Leong B, Hubbard TJP (2006). A machine learning strategy to identify candidate binding sites in human protein-coding sequence. BMC Bioinformatics.

[B14] Wang Z, Bolish ME, Yeo G, Tung V, Mawson M, Burge CB (2004). Systematic identification and analysis of exonic splicing silencers. Cell.

[B15] Fairbrother WG, Yeh RF, Sharp PA, Burge CB (2002). Predictive identification of exonic splicing enhancers in human genes. Science.

[B16] Fedorov A, Saxonov S, Fedorova L, Daizadeh I (2001). Comparison of intron-containing and intron-lacking genes elucidates putative exonic splicing enhancers. Nucleic Acids Res.

[B17] Cartegni L, Wang J, Zhu Z, Zhang MQ, Krainer AR (2003). ESEfinder: A web resource to identify exonic splicing enhancers. Nucleic Acids Res.

[B18] Pertea M, Mount SM, Salzberg SL (2007). A computational survey of candidate exonic splicing enhancer motifs in the model plant *Arabidopsis thaliana*. BMC Bioinformatics.

[B19] Cartegni L, Chew SL, Krainer AR (2002). Listening to silence and understanding nonsense: exonic mutations that affect splicing. Nat Rev Genet.

[B20] Shepelev V, Fedorov A (2006). Advances in the Exon-Intron Database (EID). Briefings in Bioinformatics.

[B21] Saxonov S, Daizadeh I, Fedorov A, Gilbert W (2000). EID: The Exon-Intron Database: An exhaustive database of protein-coding intron-containing genes. Nucleic Acids Res.

[B22] Hofacker IL, Fontana W, Stadler PF, Bonhoeffer S, Tacker M, Schuster P (1994). Fast folding and comparison of RNA secondary structures (the Vienna RNA Package). Chemical Monthly.

[B23] Gromoll J, Lahrmann L, Godmann M, Müller T, Michel C, Stamm S, Simoni M (2007). Genomic checkpoints for exon 10 usage in the luteinizing hormone receptor type 1 and type 2. Molecular Endocrinology.

